# Nanotechnology-Related Environment, Health, and Safety Research

**DOI:** 10.1289/ehp.0901029

**Published:** 2009-10

**Authors:** Giovanni Brandi, Elisabetta Nobili, Stefania Di Girolamo, Gianluca Grazi, Michelangelo Fiorentino, Rita Golfieri, Guido Biasco

**Affiliations:** S. Orsola – Malpighi Hospital, University of Bologna, Bologna, Italy, E-mail:giovanni.brandi@unibo.it

We were very interested to read the article by Schmidt (2009) about the increasing number of nanomaterials and their potential effects. We were especially interested in the similarities between carbon nanotubes and chrysotile asbestos fibers. The widespread use of asbestos-like substances with similar putative carcinogenic potential could result in the development of other unexpected types of cancer.

The potential carcinogenic risk of nano-materials that are structurally similar to asbestos and have been used in many industrial fields in the last few years has been highlighted by Carter (2008). Some studies conducted in animal models suggest that nanomaterials cause free radical–mediated cellular DNA damage and consequently have an asbestos-like carcinogenic action (Poland et al. 2008).

In addition to mesothelioma (the typical cancer index of exposure) or lung cancer, asbestos can cause other types of cancer. On the basis of our clinical experience, we hypothesize that at least a portion of bile ducts cancers (i.e., cholangiocarcinomas) are caused by exposure to this known carcinogenic agent.

From 2002 to 2008 we treated 258 patients with cholangiocarcinoma at our institute. Over the previous year, we carefully interviewed 66 consecutive patients using a standardized questionnaire asking about their exposure to asbestos and other known risk factors linked to bile duct carcino genesis (Khan et al. 2008). We collected each patient’s remote, recent, and occupational clinical history. In addition to the association with known risk factors for the onset of cholangiocarcinoma, we assessed occupational or household exposure to asbestos in 24 patients, 10 of whom did not have other certain risk factors (Table 1; Brandi et al. 2008).

Asbestos fibers cause cancer through chronic inflammation, amplifying the production of oxygen radicals, cytokines, growth factors, and proinflammatory factors responsible for both impaired antioxidant and control cell proliferation and apoptosis mechanisms in target cells (Manning et al. 2002). In contrast with findings for pleural mesothelioma, the association between exposure to asbestos and the development of other tumors, such as gastrointestinal and bile duct cancers, has not been univocally demonstrated (Gamble 1994).

The putative increased risk of bile duct cancer in subjects exposed to asbestos may be due to different mechanisms. The asbestos fibers cross the alveolar barrier by inhalation or penetrate the gastrointestinal mucosa by ingestion. They then reach the interstitial environment and circulatory system through lymphatic vessels and are finally delivered to all tissues, namely the liver and bile ducts (Miserocchi et al. 2008), where they may start a malignant transformation process (Wingren 2004). In addition, asbestos fibers may reach the bile ducts through the papilla of Vater from the intestinal lumen by retrograde reflux, as do bacteria, and remain in the gallbladder for a long time.

In the near future we may have to consider asbestos as another factor accounting for the etiopathogenesis of cholangiocarcinomas that may explain the otherwise mysterious increasing incidence of intrahepatic cholangio carcinomas in Western countries.

## Figures and Tables

**Table 1:** Characteristics of patients.

Tumor site	No. of patients	Exposure to asbestos		Possible modality of exposure		Certain added risk factors^a^	
		Household	Occupational	Ingestion	Inhalation	Absent	Present
ICC	10	7/10	4/10	7/10	4/10	6/10	4/10
Klatskin tumor	6	3/6	3/6	3/6	3/6	3/6	3/6
Ampulla of Vater	1	1/1	–	1/1	–	1/1	–
Main hepatic bile duct	1	–	1/1	–	1/1	–	1/1
GBC	6	6/6	–	6/6	–	–	6/6

Abbreviations: GBC, gallbladder carcinoma; HCV, hepatitis C virus; ICC: intrahepatic cholangiocarcinoma. Data updated from Brandi et al. (2008).

a HCV infection or exposure to chemical substances used in industry, sporadic contact with chemical substances used in farming, alcohol-related liver cirrhosis or gallstones, or ulcerative colitis and primary sclerosing cholangitis.
